# Results of Observational Studies: Analysis of Findings from the Nurses’ Health Study

**DOI:** 10.1371/journal.pone.0110403

**Published:** 2014-10-17

**Authors:** Vicky Tai, Andrew Grey, Mark J. Bolland

**Affiliations:** Department of Medicine, University of Auckland, Auckland, New Zealand; Innsbruck Medical University, Austria

## Abstract

**Background:**

The role of observational studies in informing clinical practice is debated, and high profile examples of discrepancies between the results of observational studies and randomised controlled trials (RCTs) have intensified that debate. We systematically reviewed findings from the Nurses’ Health Study (NHS), one of the longest and largest observational studies, to assess the number and strength of the associations reported and to determine if they have been confirmed in RCTs.

**Methods:**

We reviewed NHS publication abstracts from 1978–2012, extracted information on associations tested, and graded the strength of the reported effect sizes. We searched PubMed for RCTs or systematic reviews for 3 health outcomes commonly reported in NHS publications: breast cancer, ischaemic heart disease (IHD) and osteoporosis. NHS results were compared with RCT results and deemed concordant when the difference in effect sizes between studies was ≤0.15.

**Findings:**

2007 associations between health outcomes and independent variables were reported in 1053 abstracts. 58.0% (1165/2007) were statistically significant, and 22.2% (445/2007) were neutral (no association). Among the statistically significant results that reported a numeric odds ratio (OR) or relative risk (RR), 70.5% (706/1002) reported a weak association (OR/RR 0.5–2.0), 24.5% (246/1002) a moderate association (OR/RR 0.25–0.5 or 2.0–4.0) and 5.0% (50/1002) a strong association (OR/RR ≤0.25 or ≥4.0). 19 associations reported in NHS publications for breast cancer, IHD and osteoporosis have been tested in RCTs, and the concordance between NHS and RCT results was low (≤25%).

**Conclusions:**

NHS publications contain a large number of analyses, the majority of which reported statistically significant but weak associations. Few of these associations have been tested in RCTs, and where they have, the agreement between NHS results and RCTs is poor.

## Introduction

Observational research is commonly undertaken, reported and publicised, but the role of observational studies in informing clinical practice is debated. High quality randomised controlled trials (RCTs) are usually considered to be the highest level of evidence (Level 1), with high quality cohort studies ranked immediately below this (Level 2) [1]. Some authors have suggested a broad role for observational studies because the study population may better represent the general population than in RCTs, because RCTs can be difficult or impossible to carry out for some conditions, and because systematic reviews have generally reported that results from observational studies do not differ markedly from RCTs [2]–[5]. However, a number of high profile examples of discrepancies between results of observational studies and subsequent RCTs have led others to suggest the role for observational studies should be limited [6]–[8]. Observational studies suggested beneficial effects of oestrogen with progesterone on cardiovascular disease [9], antioxidants on cancer prevention [10], and folic acid/B vitamins for cardiovascular disease [11], but subsequent RCTs reported either harms [12]–[15] or no benefits [16]–[18] from these agents. Because observational studies cannot test causality, one view is that their results should be regarded as hypothesis-generating and should not influence clinical practice until these hypotheses are tested in adequately powered RCTs [19]. Others suggest that small effects seen in observational studies should not be considered credible because they are more likely to represent bias and confounding than a causal relationship [8].

One of the largest, longest and most influential observational studies is the Nurses’ Health Study (NHS). The NHS began in 1976 and has subsequently followed more than 100,000 women in the original cohort study. Numerous papers in high impact biomedical journals have originated from this study. The size, duration and eminence of the NHS make it a good model to formally explore the scope, veracity and impact of data from observational analyses. In the present work, we have undertaken a systematic review of publications from the NHS. We set out to determine how many hypotheses have been explored in NHS publications, the strength of the associations reported, and how these findings align with those from RCTs on the same topics.

## Methods

### NHS publications

**Figure 1:**
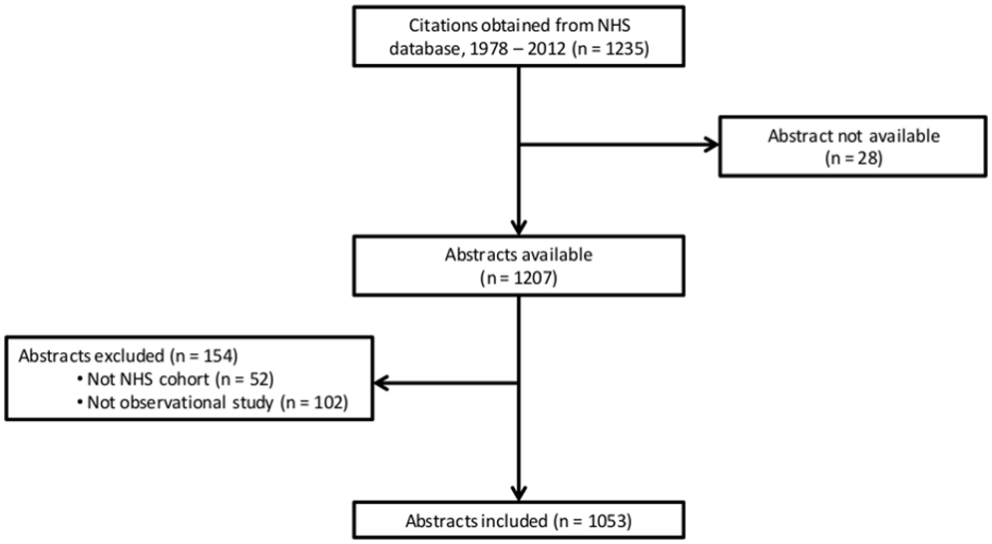
Flowchart of study selection.

In November 2013, we extracted the citations of all 1235 NHS publications between 1978–2012 from the NHS website (http://www.channing.harvard.edu/nhs/?page_id=154). We included publications with an abstract, those in which the NHS cohort was part of the population studied, and those with an observational (case-control or cohort) study design. Figure 1 shows the flow of studies. 28 publications did not have an abstract, 52 studies did not include the NHS cohort, and 102 publications did not report findings from observational analyses, leaving 1053 publications included in our analyses.

One investigator (VT) reviewed the abstracts of all eligible publications. For all analyses reported in the abstracts, we extracted information on the associations analysed by the investigators, the endpoints assessed, the independent variables for each of those endpoints, and the reported effect sizes and 95% confidence intervals (CIs). We classified each result by the strength and direction of the association reported and the level of statistical significance. Statistically significant results with an odds ratio (OR) or relative risk (RR) of ≤0.25 or ≥4 were considered strong associations, those with OR/RR of 0.25–0.5 or 2–4 were considered moderate associations, and those with OR/RR of 0.5–2 were considered weak associations [8]. When the CI for the OR/RR of a reported association included 1 but the text implied a relationship between the outcome and independent variable existed, we classified these associations as statistically non-significant.

### Randomised clinical trials

We selected 3 health outcomes (breast cancer, ischaemic heart disease [IHD] and osteoporosis) that are important to women’s health and were frequently studied in NHS publications. One investigator (VT) searched PubMed for RCTs or systematic reviews of RCTs with breast cancer, IHD and osteoporosis as the primary endpoint that evaluated similar factors to the independent variables studied in NHS publications. We used the following format in our PubMed search: health outcome AND independent variable AND random*. We included the latest meta-analysis of RCTs identified, and when one was not available or suitable, we included all large relevant RCTs. Two investigators (VT and MB) reviewed the full texts of these meta-analyses or RCTs and extracted information on effect sizes and 95% CIs for the individual result or the pooled analyses of RCTs.

### Comparison of results of NHS publications with RCT results

We compared the effect sizes reported in the NHS publications with those from relevant RCTs, and considered them concordant when the difference between the effect sizes was ≤0.15. There is no generally accepted definition of concordance of results from studies of different designs. We chose a threshold of an absolute difference of 15% on the basis that this effect size is close to the smallest that is clinically meaningful. Smaller effect sizes are generally unlikely to be considered clinically meaningful to patients, because the absolute benefits from taking a treatment are small in this situation.

## Results

### NHS results

**Figure 2:**
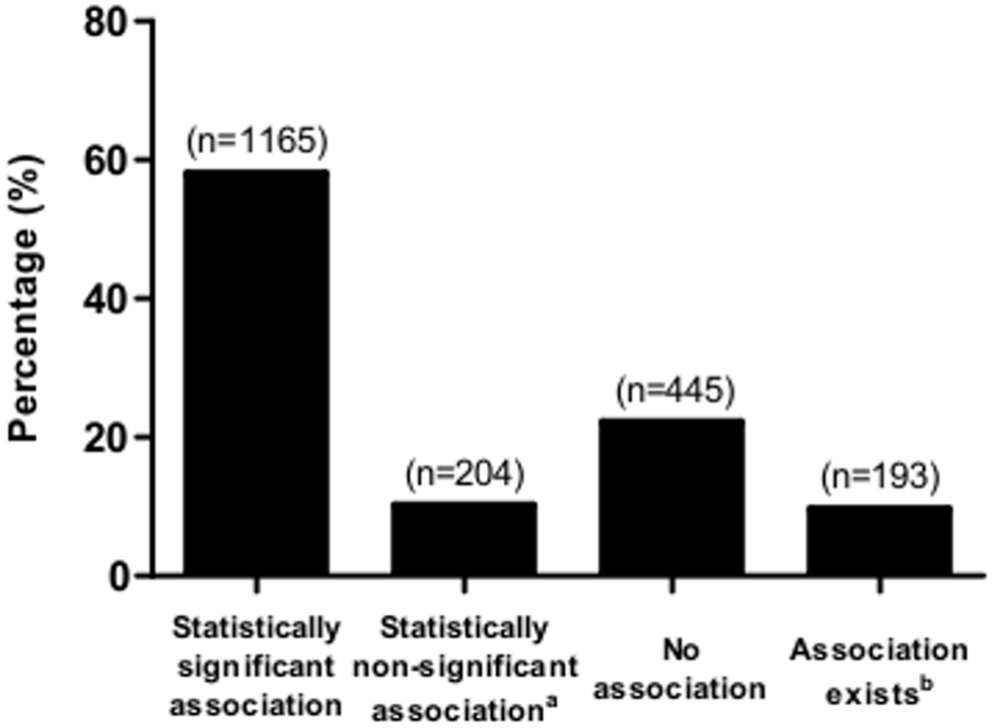
Results of 2007 tests of associations from 1053 NHS publications. ^a^Results were statistically non-significant but the abstract implied an association exists. ^b^An association was reported in the abstract with insufficient information to determine the strength or direction of the association.

**Figure 3:**
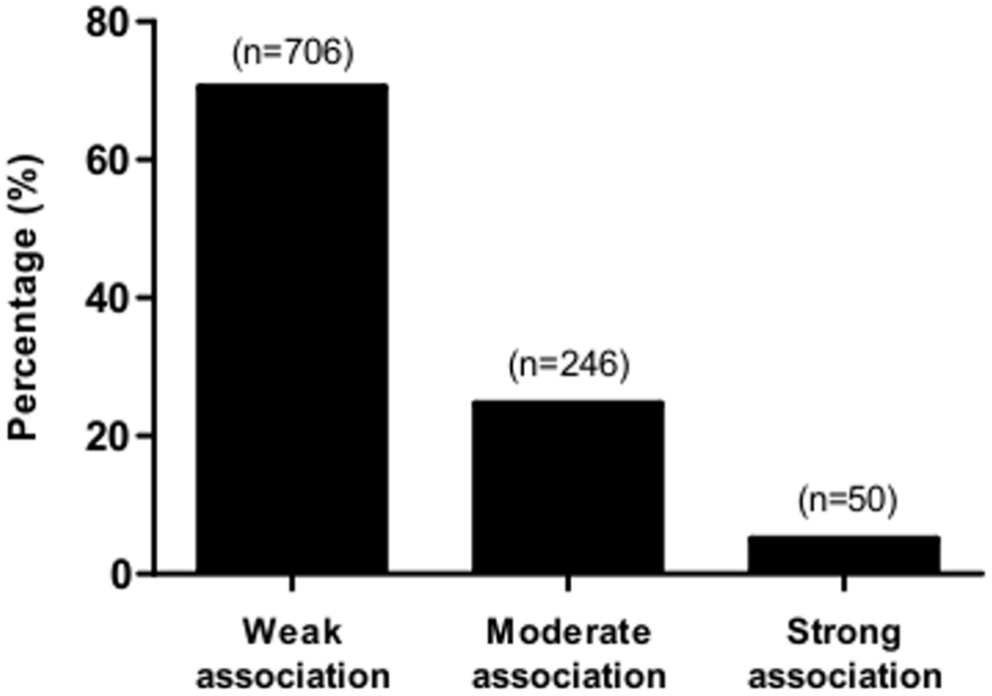
Strength of 1002 statistically significant relative risks and odds ratios reported in NHS publications. Associations with an odds ratio (OR) or relative risk (RR) of ≤0.25 or ≥4 were considered strong, those with OR/RR of 0.25–0.5 or 2–4 were considered moderate, and those with OR/RR of 0.5–2 were considered weak.

Associations between 61 health outcomes and 1383 independent variables were reported in the abstracts of 1053 NHS publications (Table S1). Many of these independent variables were reported slightly differently or were closely related in different publications and so we were able to classify them into 136 broad groups comprising closely related variables. The three most commonly tested outcomes were breast cancer, colorectal cancer and IHD, and associations were reported with these endpoints for 56, 49 and 46 broad groups of independent variables, respectively (Table 1). In total, 2007 associations between health outcomes and independent variables were reported. Of these associations, 1433 (71.4%) were results from the NHS cohort alone, and 574 (28.6%) were from studies where the NHS cohort was pooled with other cohorts. Figure 2 shows that 1165 (58.0%) of the 2007 associations reported were statistically significant (477 beneficial, 688 harmful), 204 (10.2%) were statistically non-significant but the abstract implied an association exists (114 beneficial, 90 harmful), and 445 (22.2%) were neutral (no association). The majority of the 204 statistically non-significant results reported effect sizes for an individual subgroup that was not statistically significant, but a test for a trend across the subgroups was statistically significant. For a further 193 (9.6%) results, an association was reported in the abstract but there was insufficient information to determine whether the association was beneficial or harmful. Among the 1165 statistically significant associations, 1002 had a reported numeric RR or OR. Figure 3 shows that the majority of these associations, 706 (70.5%), were weak, with 246 (24.5%) associations classified as moderate, and only 50 (5.0%) classified as strong associations.

**Table 1:** Results of 2007 associations reported in 1053 Nurses’ Health Study Abstracts.

Outcome	Publications (n)	Individual	Broad groups	Statistically significant		Statistically		No	Association
		independent	of independent	association		non-significant		association	exists^c^
		variables (n)^a^	variables (n)	(n)		association^b^ (n)		(n)	(n)
				Harmful	Beneficial	Harmful	Beneficial		
**All Outcomes**	1053	1383	136	688	477	90	114	445	193
**Individual outcomes**									
Breast cancer	209	326	56	119	56	17	23	134	28
Colorectal cancer	123	207	49	47	59	11	21	38	33
Ischaemic heart disease	98	137	46	54	48	8	6	13	12
Diabetes mellitus	55	95	32	47	32	3	1	8	4
Serum markers	51	119	32	40	38	1	0	1	42
Ovarian cancer	50	95	35	24	17	9	10	41	3
Mortality	43	57	21	28	23	0	1	2	4
Cognitive ability	31	46	19	17	10	0	4	10	5
Colorectal adenoma	30	58	24	16	13	3	7	17	2
Endometrial cancer	29	53	21	15	13	1	5	18	1
Skin cancer	29	54	14	25	5	7	2	11	4
Stroke	28	52	26	24	20	2	5	0	1
Cardiovascular disease	25	36	22	16	12	2	2	3	2
Pancreatic cancer	24	44	19	18	3	2	1	20	1
Kidney disease	23	42	27	20	12	1	3	7	1
Hypertension	22	31	18	17	12	0	0	2	0
Cataract	21	34	16	10	15	3	3	3	0
Osteoporosis	19	33	19	10	8	2	1	9	3
Rheumatoid arthritis	18	33	19	7	1	7	0	17	1
Body weight	18	25	16	11	5	0	0	2	7
Gallstones	15	24	15	9	11	0	1	3	1
Parkinson’s disease	13	24	17	1	7	2	4	9	1
Urinary incontinence	12	25	10	17	3	0	0	3	2
General Health	11	19	5	10	2	0	0	0	9
Lung cancer	11	18	11	1	7	1	2	6	2
Macular degeneration	11	16	10	8	4	0	0	3	1
Cancer- general	10	13	8	7	1	0	0	4	1
Glaucoma	9	19	13	2	4	0	0	13	0
Kidney cancer	9	20	13	5	3	0	6	5	2
Mental health	9	14	9	6	5	1	0	1	1
Asthma	7	11	9	5	2	0	1	0	3
Chronic disease	7	7	3	1	5	0	0	1	1
Mammographic density	7	13	2	4	3	0	0	7	0
Sudden cardiac death	7	7	7	2	4	1	0	0	0
Bladder cancer	5	7	7	2	0	0	0	5	0
Thrombotic disease	5	8	8	5	0	1	0	2	0
Brain tumor	5	12	9	3	1	1	0	6	1
Systemic lupus erythematosus	5	5	5	1	0	1	0	3	0
Lymphoma	4	8	6	3	1	1	2	0	1
Menarche	4	3	2	0	0	0	0	1	3
Menopause	4	3	3	1	0	1	0	0	3
Telomere length	4	7	6	3	3	0	0	1	0
Urine composition	4	13	6	8	4	0	0	1	0
Dental health	3	4	3	0	0	0	0	2	2
Dietary quality	3	4	3	1	0	0	0	1	2
Gout	3	9	7	5	4	0	0	0	0
Multiple sclerosis	3	7	3	0	0	1	1	5	0
Smoking	3	3	3	1	0	0	0	1	1
Amyotrophic lateral sclerosis	2	4	2	2	0	0	2	0	0
Chronic obstructive pulmonary disease	2	2	2	1	0	0	0	1	0
Ageing	1	1	1	0	1	0	0	0	0
Barrett’s esophagus	1	2	1	1	0	0	0	1	0
Bronchitis	1	1	1	1	0	0	0	0	0
Caffeine intake	1	1	1	0	0	0	0	0	1
Complementary Medicine	1	3	1	0	0	0	0	3	0
Connective tissue disease	1	1	1	0	0	0	0	1	0
Falls	1	1	1	1	0	0	0	0	0
Hair colour/skin pigmentation	1	1	1	0	0	0	0	0	1
Immunologic disease	1	1	1	1	0	0	0	0	0
Insulin resistance	1	2	1	2	0	0	0	0	0
Osteoarthritis	1	3	2	3	0	0	0	0	0

a In total 2007 associations were reported between 61 health outcomes and 1383 independent variables.

b Results were statistically non-significant but the abstract implied an association exists.

c An association was reported in the abstract with insufficient information to determine the strength or direction of the association.

Table 2 shows the journals and frequency of NHS publications. The impact of NHS publications is apparent: 30% of the publications were published in journals with impact factors >10, and 15% were published in one of the 6 most prestigious internal medicine journals (Annals of Internal Medicine, Archives of Internal Medicine, BMJ, Lancet, JAMA, New England Journal of Medicine).

**Table 2:** Top 20 most frequently published Journals for Nurses’ Health Study publications.

Journal	Impact factor	Number of publications
Cancer Epidemiology Biomarkers and Prevention	4.6	81
American Journal of Epidemiology	4.8	80
American Journal of Clinical Nutrition	6.5	66
Journal of the National Cancer Institute	14	59
Journal of the American Medical Association	30	50
International Journal of Cancer	6.2	49
New England Journal of Medicine	52	38
Cancer Causes and Control	3.2	35
Archives of Internal Medicine	11	33
Cancer Research	8.7	25
Diabetes Care	7.7	23
Circulation	15	22
Annals of Internal Medicine	14	20
CA-A Cancer Journal for Clinicians	153	18
Journal of Clinical Oncology	18	15
Nature Genetics	35	13
Breast Cancer Research	5.9	12
Carcinogenesis	5.6	12
Human Molecular Genetics	7.7	12
PLoS One	3.7	12

### Comparisons between NHS findings and RCT results

The results from NHS publications and relevant RCTs are summarised in Table 3 for breast cancer, Table 4 for IHD, and Table 5 for osteoporosis. Of the 49 associations in NHS publications for these 3 outcomes, 16 were not statistically significant, and 30 statistically significant associations were classified as weak, 3 as moderate, and 0 as strong. For breast cancer, NHS publications reported associations with 56 broad groups of predictive factors. Of these factors, 8 have been tested in RCTs [12], [13], [20]–[27], and 6/24 (25%) of effect sizes in NHS publications [28]–[43] were concordant with those from RCTs. For IHD, NHS publications reported associations with 46 broad groups of predictive factors, of which 7 have been tested in RCTs [44]–[47]. 2/19 (10.5%) of effect sizes in NHS publications [48]–[60] were concordant with those from RCTs. For osteoporosis, NHS publications reported associations with 19 broad groups of predictive factors, of which 4 have been tested in RCTs [61]–[64]. 1/5 (20%) of effect sizes in NHS publications [65]–[68] were concordant with those from RCTs. Of the 39 discordant results for these 3 endpoints, 29 NHS results were more positive (ie a smaller RR or OR) than the RCT results. 8 of the 10 discordant results where the NHS results were more negative (ie a larger RR or OR) than the RCT results were from NHS publications examining the relationship between oestrogen and breast cancer.

**Table 3:** Results of Nurses’ Health Study and related randomised clinical trials for breast cancer.

Randomised controlled trials			Nurses’ Health Study Publications			
Study	Description	Effect size	Study	Description	Effect size	Concordance
		(95% CI)			(95% CI)	
Calcium			Calcium			
Bristow 2013 [20]	Meta-analysis of 6 RCTs	1.01	Shin 2002[28]	Cohort study	0.69	No
	V: Calcium supplements	(0.64–1.59)		V: Dairy calcium intake	(0.48–0.98)	
	O: Breast cancer			O: Premenopausal breastcancer		
**Beta-carotene**			**Beta-carotene**			
Druesne-Pecollo	Meta-analysis of 4 RCTs	0.96	Tamimi 2005[29]	Case-control study	0.73	No
2009 [21]	V: Beta-carotenesupplementation	(0.85–1.10)		V: Plasma beta-carotene	(0.53–1.02)	
	O: Breast cancer			O: Breast cancer		
			Zhang 2012[30]	Pooled analysis of 18 cohortstudies	0.84	Yes
				V: Beta-carotene intake	(0.77–0.93)	
				O: ER-negative breast cancer		
**Folate**			**Folate**			
Vollset 2013 [22]	Meta-analysis of 13 RCTs	0.89	Zhang 1999[31]	Cohort study	0.55	No
	V: Folic acidsupplementation	(0.66–1.20)		V: Folate intake	(0.39–0.76)	
	O: Breast cancer			O: Breast cancer		
			Zhang 2005[32]	Cohort study	1	Yes
				V: Folate intake	(0.89–1.14)	
				O: ER-positive breast cancer		
			Zhang 2005[32]	Cohort study	0.81	Yes
				V: Folate intake	(0.66–0.99)	
				O: ER-negative breast cancer		
**Aspirin**			**Aspirin**			
Cook 2005 [23]	Women’s Health Study RCT	0.98	Egan 1996[33]	Cohort study	1.03	Yes
	V: Low-dose aspirin	(0.87–1.09)		V: ≥2 tablets aspirin/week	(0.95–1.12)	
	O: Breast cancer			O: Breast cancer		
			Holmes 2010[34]	Cohort study of stages 1–3 breastcancer	0.36	No
				V: Aspirin 6–7 days/week	(0.24–0.54)	
				O: Breast cancer mortality		
			Holmes 2010[34]	Cohort study of stages 1–3 breastcancer	0.57	No
				V: Aspirin 6–7 days/week	(0.39–0.82)	
				O: Distant recurrence		
			Holmes 2011[35]	Cohort study of COX-2-positivebreast cancer	0.64	No
				V: Aspirin	(0.43–0.96)	
				O: Breast cancer mortality		
			Holmes 2011[35]	Cohort study of COX-2-positivebreast cancer	0.57	No
				V: Aspirin	(0.44–0.74)	
				O: Distant recurrence		
**Postmenopausal hormones – oestrogen**			**Postmenopausal hormones – oestrogen**			
Anderson 2004[24]	Women’s HealthInitiative RCT	0.77	Colditz 1990[36]	Cohort study	1.36	No
	V: Oestrogen	(0.59–1.01)		V: Oestrogen	(1.11–1.67)	
	O: Breast cancer			O: Breast cancer		
			Colditz 1992[37]	Cohort study	1.42	No
				V: Oestrogen	(1.19–1.70)	
				O: Breast cancer		
			Colditz 1995[38]	Cohort study	1.32	No
				V: Oestrogen	(1.14–1.54)	
				O: Breast cancer		
			Colditz 2000[39]	Cohort study	1.23	No
				V: Oestrogen use from ages50–60 y	(1.06–1.42)	
				O: Cumulative risk of breastcancer to age 70 y		
			Chen 2006[40]	Cohort study among women withhysterectomy	1.42	No
				V: Oestrogen use for ≥20 y	(1.13–1.77)	
				O: Breast cancer		
			Chen 2006[40]	Cohort study among women withhysterectomy	1.48	No
				V: Oestrogen use for ≥15 y	(1.05–2.07)	
				O: ER-positive/PR-positive breastcancer		
**Postmenopausal hormones – oestrogen + progestin**			**Postmenopausal hormones – oestrogen + progestin**			
Hulley 1998[12]	Heart andoestrogen/progestin	1.3	Colditz 1992[37]	Cohort study	1.54	No
	replacement study RCT	(0.77–2.19)		V: Oestrogen + progestin	(0.99–2.39)	
	V: Oestrogen + progestin			O: Breast cancer		
	O: Postmenopausal breastcancer					
Rossouw 2002[13]	Women’s Health InitiativeRCT	1.26	Colditz 1995[38]	Cohort study	1.41	Yes
	V: Oestrogen + progestin	(1.00–1.59)		V: Oestrogen + progestin	(1.15–1.74)	
	O: Postmenopausal breastcancer			O: Breast cancer		
			Colditz 2000[39]	Cohort study	1.67	No
				V: Oestrogen + progestin	(1.18–2.36)	
				O: Cumulative risk of breastcancer to age 70 y		
**Statins**			**Statins**			
Pfeffer 2002[25]	3 RCTs	2	Eliassen 2005[41]	Cohort study	0.91	No
	V: Pravastatin	(0.97–4.11)		V: Statins	(0.76–1.08)	
	O: Breast cancer			O: Breast cancer		
**Vitamin D**			**Vitamin D**			
Sperati 2013[26]	Meta-analysis of 2 RCTs	1.11	Shin 2002[28]	Cohort study	0.72	No
	V: Vitamin D supplements	(0.74–1.68)		V: Vitamin D intake	(0.55–0.94)	
	O: Breast cancer			O: Premenopausal breastcancer		
			Bertone-Johnson	Case-control study	0.73	No
			2005 [42]	V: Plasma levels of25-hydroxyvitamin D	(0.49–1.07)	
				O: Breast cancer		
**Vitamin E**			**Vitamin E**			
Lee 2005[27]	Women’s Health Study RCT	1	Hunter 1993[43]	Cohort study	0.99	Yes
	V: Vitamin E supplements	(0.90–1.12)		V: Vitamin E intake	(0.83–1.19)	
	O: Breast cancer			O: Breast cancer		

CI = confidence interval; RCT = randomised controlled trial; V = independent variable; O = health outcome or endpoint; ER = oestrogen receptor; PR = progesterone receptor.

**Table 4:** Results of Nurses’ Health Study and related randomised clinical trials for ischaemic heart disease.

Randomised controlled trials		Nurses’ Health Study Publications			
Study	Effect size	Study	Description	Effect size	Concordance
	(95% CI)			(95% CI)	
Beta-carotene		Beta-carotene			
Myung	0.96	Osganian	Cohort study	0.74	No
2013 [44]	(0.92–1.04)	2003 [48]	V: Beta-carotene intake	(0.59–0.93)	
			O: Coronary artery disease		
**Omega-3 fatty acids**		**Omega-3 fatty acids**			
Kotwal	0.86	Hu 1999 [49]	Cohort study	0.55	No
2012 [45]	(0.67–1.11)		V: Alpha-linolenic acid intake	(0.32–0.94)	
			O: Fatal ischaemic heart disease		
					
		Hu 2002 [50]	Cohort study	0.67	No
			V: Omega-3 fatty acid intake	(0.55–0.81)	
			O: Coronary heart disease		
		Hu 2003 [51]	Cohort study of type 2 diabetes	0.69	No
			V: Long-chain omega-3 fatty acid intake	(0.47–1.03)	
			O: Coronary heart disease		
**Folate**		**Folate**			
Myung	0.99	Rimm 1998 [52]	Cohort study	0.69	No
2013 [44]	(0.95–1.02)		V: Folate intake	(0.55–0.87)	
			O: Coronary heart disease		
**Aspirin**		**Aspirin**			
Berger	0.86	Manson	Cohort study	0.75	Yes
2011 [46]	(0.74–1.00)	1991 [53]	V: 1–6 aspirin/week	(0.58–0.99)	
			O: Myocardial infarction		
**Postmenopausal hormones – oestrogen**		**Postmenopausal hormones – oestrogen**			
Yang	0.93	Bain 1981 [54]	Case-control study	0.7	No
2013 [47]	(0.80–1.08)		V: Oestrogen	(0.5–1.1)	
			O: Myocardial infarction		
Yang	0.95	Stampfer	Cohort study	0.3	No
2013 [47]	(0.78–1.15)	1985 [55]	V: Estorgen	(0.2–0.6)	
			O: Coronary disease		
		Stampfer	Cohort study	0.56	No
		1991 [56]	V: Oestrogen	(0.40–0.80)	
			O: Coronary disease		
		Grodstein	Cohort study	0.61	No
		2000 [57]	V: Hormone therapy - oestrogen^a^	(0.52–0.71)	
			O: Coronary events		
		Grodstein	Cohort study	0.54	No
		2000 [57]	V: 0.625 mg/d oral conjugated oestrogen	(0.44–0.67)	
			O: Coronary events		
		Grodstein	Cohort study of previous coronary disease	1.25	No
		2001 [58]	V: Short-term use of oestrogen	(0.78–2.00)	
			O: Recurrent coronary heart disease events		
		Grodstein	Cohort study of previous coronary disease	0.38	No
		2001 [58]	V: Long-term use of oestrogen	(0.22–0.66)	
			O: Recurrent coronary heart disease events		
		Grodstein	Cohort study	0.66	No
		2006 [59]	V: Oestrogen (beginning near menopause)	(0.54–0.80)	
			O: Coronary heart disease		
		Grodstein	Cohort study	0.87	Yes
		2006 [59]	V: Oestrogen (beginning >10 yafter menopause)	(0.69–1.10)	
			O: Coronary heart disease		
**Postmenopausal hormones –** **oestrogen + progestin**		**Postmenopausal hormones – oestrogen + progestin**			
Yang	1.07	Grodstein	Cohort study	0.72	No
2013 [47]	(0.91–1.26)	2006 [59]	V: Oestrogen + progestin(beginning near menopause)	(0.56–0.92)	
			O: Coronary heart disease		
Yang	1.09	Grodstein	Cohort study	0.9	No
2013 [47]	(0.85–1.41)	2006 [59]	V: Oestrogen + progestin (beginning >10 y	(0.62–1.29)	
			after menopause)		
			O: Coronary heart disease		
**Vitamin B**		**Vitamin B**			
Myung	0.96	Rimm 1998 [52]	Cohort study	0.67	No
2013 [44]	(0.92–1.01)		V: Vitamin B6 intake	(0.53–0.85)	
			O: Coronary heart disease		
**Vitamin E**		**Vitamin E**			
Myung	0.97	Stampfer	Cohort study	0.66	No
2013 [44]	(0.94–1.01)	1993 [60]	V: Vitamin E intake	(0.50–0.87)	
			O: Major coronary disease		

a The type of hormone therapy was not described in the paper, but is most likely to be oestrogen without progesterone.

CI = confidence interval; RCT = randomised controlled trial; V = independent variable; O = health outcome or endpoint.

**Table 5:** Results of Nurses’ Health Study and related randomised clinical trials for osteoporosis.

Randomised controlled trials			Nurses’ Health Study Publications			
Study	Description	Effect size	Study	Description	Effect size	Concordance
		(95% CI)			(95% CI)	
Calcium			Calcium			
Reid 2014 [61]	Meta-analysis of 5 RCTs	1.61	Feskanich	Cohort study	1.45	No
	V: Calcium supplements	(0.91–2.85)	1997 [65]	V: Calcium intake	(0.87–2.43)	
	O: Hip fracture			O: Hip fracture		
			Feskanich	Cohort study	0.96	No
			2003 [66]	V: ≥1200 mg/d total calciumintake	(0.68–1.34)	
				O: Hip fracture		
**Fluoride**			**Fluoride**			
Vestergaard	Meta-analysis of 8 RCTs	0.8	Feskanich	Case-control study	0.8	Yes
2008 [62]	V: Fluoride formulations	(0.5–1.4)	1998 [67]	V: Toenail fluoride	(0.2–4.0)	
	O: Non-vertebral fracture			O: Hip fracture		
			Feskanich	Case-control study	1.6	No
			1998 [67]	V: Toenail fluoride	(0.8–3.1)	
				O: Forearm fracture		
**Vitamin D**			**Vitamin D**			
Avenell 2009 [63]	Meta-analysis of 9 RCTs	1.15	Feskanich	Cohort study	0.63	No
	V: Vitamin D supplements	(0.99–1.33)	2003 [66]	V: ≥12.5 mcg/d Vitamin Dintake	(0.42–0.94)	
	O: Hip fracture			O: Hip fracture		
**Vitamin K**			**Vitamin K**			
Stevenson	Meta-analysis of 3 RCTs	0.27	Feskanich	Cohort study	0.7	N/A^a^
2009 [64]	V: Vitamin K2 supplements	(0.03–2.38)^a^	1999 [68]	V: Vitamin K intake	(0.53–0.93)	
	O: Hip fracture			O: Hip fracture		

a Based on 3 hip fractures only in RCTS. Therefore, insufficient data for comparison between studies.

CI = confidence interval; RCT = randomised controlled trial; V = independent variable; O = health outcome or endpoint; N/A = Not available.

## Discussion

NHS publications report a very large number of associations between health outcomes and independent variables. Only 1 in 5 associations was reported as neutral (no association). Of the statistically significant associations, only 5% were strong associations (OR/RR ≤0.25 or ≥4), with 70% of effect sizes being weak (OR/RR between 0.5 and 2.0). Few of the associations have been tested in RCTs and, where relevant RCTs have been reported, only 1 in 5 NHS study results was concordant with the RCT result. Despite this, NHS publications were frequently published in high impact journals.

More than 2000 associations from this single study were reported in the abstracts of publications we reviewed. This is likely to be a substantial underestimate of the actual number of associations examined, because many results will have only been reported in the text or tables of the full article or will not have been reported. The large number of statistical tests raises concerns about false positive results. None of the abstracts highlighted this possibility, reported analyses adjusted for multiple statistical testing, or mentioned the number of analyses previously conducted in the NHS cohort.

1358 results (68%) in NHS publication abstracts were either statistically significant or reported as though an association existed. It is difficult to estimate the likely number of false positives amongst these results. If all of the 2007 associations examined were of unrelated variables and there was no relationship between the health outcomes and these variables, about 100 results (5%) would be statistically significant due to chance. However, many of the variables examined were closely related which would decrease the total number of independent tests. On the other hand, it is likely that the results reported in the abstract are only a small proportion of the total statistical tests conducted (either reported in the full article or not reported) which would substantially increase the total number of independent tests. Furthermore, statistically significant results are more likely to be reported in the abstract than non-significant results. Given the likely bias toward significant results and the very large number of statistical tests performed, it seems reasonable to conclude that a substantial proportion of results were false positives. This concern was not raised in any of the abstracts.

The strength of associations reported in observational studies is often viewed as an indicator of the credibility of the association [8], [69]–[71]. Associations with OR or RR ≥4 or ≤0.25 are considered strong and more likely to be reliable in the absence of significant bias [8], [70], [71]. However, where the association is weak or moderate, such results should be viewed with scepticism. Effect sizes may be inflated, observational studies are limited by selection bias, confounding, and methodological weaknesses in their study design and analysis, and large observational studies can produce implausibly precise estimates of effect sizes that are highly statistically significant but clinically unimportant [8], [69]–[71]. Only 5% of results reported in NHS publications were strong associations. Despite this, a very large number of NHS papers were published in high-impact general medical and speciality journals. A recent survey reported that only 14% of publications of observational studies in high impact medical journals called for RCTs to support their findings, with the majority making explicit recommendations regarding clinical practice based upon the observational study findings [19]. Taken together, these findings suggest that many journals, including high impact journals, place a low importance on the strength of an association or the non-randomised nature of the study and hence the credibility of the association when evaluating observational studies for publication. In addition, since clinical research findings published in prominent journals influence clinical behaviour, our findings suggest that clinical practice might often be driven by false positive results from observational studies.

We compared findings from NHS publications and RCTs for 3 important health outcomes that were studied commonly in NHS publications. Results of 496 associations between breast cancer, IHD, and osteoporosis and independent variables were reported in 326 publications. However, few RCTs examining the relationship between these outcomes and the independent variables have been undertaken. Thus, we identified RCTs for only 19 of these broad groups of variables for these 3 outcomes, and the concordance between the results of the RCTs and the NHS results was poor. The reasons for the small number of RCTs are not clear. It is possible that investigators do not view the NHS results as credible because of the small effect sizes, and thus have not chosen to examine their findings in RCTs, but this seems unlikely. A possible explanation is that RCTs are more difficult, more expensive, and take longer to conduct than new analyses of the NHS, or comparable analyses of other observational datasets. In addition, the volume of hypotheses generated – about 30 NHS papers eligible for our analysis were published annually – and the small effect sizes reported means that an impractically large number of very large RCTs would be needed to test all the associations reported. About 60% of associations reported by NHS studies suggested a harmful effect of the independent variable on the outcome. This is another possible explanation for the small number of RCTs as directly assessing potential harms in an RCT is likely to be unattractive to researchers, ethics committees, funding bodies, and participants. However, potential harms identified in observational studies can usually be indirectly assessed in RCTs, by exploring whether interventions that reduce the potential harmful exposure improve health outcomes. If reduction of a potentially harmful exposure has no impact on health outcomes, this suggests that harm from the exposure is spurious and not clinically relevant.

Previous systematic comparisons of the results of observational studies and RCTs have reported that pooled results from observational studies generally do not differ markedly from pooled results from RCTs [2]–[4]. However, within these pooled analyses, there were marked variations in individual results, discrepancies did occur, and differences in estimated magnitude of treatment effect were common [3], [4]. There was agreement between the results of NHS publications and relevant RCTs for only 10–25% of analyses for the 3 outcomes we assessed. The low rate of concordance likely reflects the propensity of observational analyses to generate inaccurate estimates of effect, as a result of confounding and bias. Other contributing factors might be that our definition of concordance was quite stringent, or that the factors studied in RCTs were not always identical to those studied in NHS publications (eg. calcium supplements vs. dietary calcium intake).

In summary, we found that a very large number of associations have been reported in NHS publications, but 95% were weak or moderate in strength, and therefore unlikely to be causal. Few of these associations have been tested in RCTs, and where they have been, agreement between NHS and RCT findings is poor. Clinicians interpreting the findings of observational studies such as the NHS should be aware of the possibility that multiple statistical tests have been undertaken with the resulting likelihood of false positive results, and of the lack of credibility for associations where the effect size is small. The low concordance of NHS findings with RCT findings suggests that clinical practice should not be informed by observational studies, and that findings from observational studies should not necessarily lead to confirmatory RCTs being conducted, especially when the effect size is small. Reporting of observational studies would be improved by including the total number of associations ever tested in the study, the proportions of statistically significant results previously published, and whether previous findings from the observational study are concordant with RCTs.

## Supporting Information

Table S1 **Database of information extracted from 1053 NHS publication abstracts.**
(XLSX)
